# Human iPSC-derived iMSCs improve bone regeneration in mini-pigs

**DOI:** 10.1038/s41413-019-0069-4

**Published:** 2019-10-24

**Authors:** Pascal Jungbluth, Lucas-Sebastian Spitzhorn, Jan Grassmann, Stephan Tanner, David Latz, Md Shaifur Rahman, Martina Bohndorf, Wasco Wruck, Martin Sager, Vera Grotheer, Patric Kröpil, Mohssen Hakimi, Joachim Windolf, Johannes Schneppendahl, James Adjaye

**Affiliations:** 1 Department of Trauma and Hand Surgery, Heinrich Heine University Hospital Düsseldorf, Moorenstr. 5, 40225 Düsseldorf, Germany; 2 Institute for Stem Cell Research and Regenerative Medicine, Medical Faculty, Heinrich Heine University, Düsseldorf, Moorenstr. 5, 40225 Düsseldorf, Germany; 3 Animal Research Institute, Heinrich Heine University, Düsseldorf, Moorenstr. 5, 40225 Düsseldorf, Germany; 4 Department of Diagnostic and Interventional Radiology, Medical Faculty, Heinrich Heine University, Düsseldorf, Moorenstr. 5, 40225 Düsseldorf, Germany; 5 Vivantes Klinikum Am Urban, Dieffenbachstraße 1, 10967 Berlin, Germany

**Keywords:** Bone, Bone quality and biomechanics

## Abstract

Autologous bone marrow concentrate (BMC) and mesenchymal stem cells (MSCs) have beneficial effects on the healing of bone defects. To address the shortcomings associated with the use of primary MSCs, induced pluripotent stem cell (iPSC)-derived MSCs (iMSCs) have been proposed as an alternative. The aim of this study was to investigate the bone regeneration potential of human iMSCs combined with calcium phosphate granules (CPG) in critical-size defects in the proximal tibias of mini-pigs in the early phase of bone healing compared to that of a previously reported autograft treatment and treatment with a composite made of either a combination of autologous BMC and CPG or CPG alone. iMSCs were derived from iPSCs originating from human fetal foreskin fibroblasts (HFFs). They were able to differentiate into osteoblasts in vitro, express a plethora of bone morphogenic proteins (BMPs) and secrete paracrine signaling-associated cytokines such as PDGF-AA and osteopontin. Radiologically and histomorphometrically, HFF-iMSC + CPG transplantation resulted in significantly better osseous consolidation than the transplantation of CPG alone and produced no significantly different outcomes compared to the transplantation of autologous BMC + CPG after 6 weeks. The results of this translational study imply that iMSCs represent a valuable future treatment option for load-bearing bone defects in humans.

## Introduction

The majority of bone fractures heal without complications. However, cases involving bone nonunion and large skeletal bone defects represent a challenge for orthopedic surgery. Despite its significant drawbacks, including donor site morbidity, limited availability, and poor bone quality, autologous bone grafting remains the gold standard for treatment.^1^ The use of autologous bone marrow concentrate (BMC) or mesenchymal stem cells (MSCs) have been described as alternative treatment options for improving bone regeneration.^2,3^

BMC contains stem cells, growth factors, and immune cells and have been shown to improve bone regeneration.^4^ MSCs are multipotent, which is manifested in their ability to differentiate into adipocytes, chondrocytes and osteoblasts in vitro.^5–8^

MSCs, as well as BMC, have been used in large animal studies for bone regeneration in weight-bearing and nonweight-bearing conditions.^4,9^ However, the availability of MSCs is restricted and associated with complications such as donor site comorbidity related to the invasive isolation from bone marrow or other tissues such as fat.^10^ Furthermore, it has been demonstrated that their differentiation and proliferation capacity decreases with donor age and the duration of culture.^11–13^

MSCs differentiated from embryonic or induced pluripotent stem cells (ESCs, iPSCs), termed iMSCs, represent an alternative to primary MSCs. As the use of ESCs is associated with ethical concerns, iPSC-derived iMSCs have been identified as a promising source of transplantable donor cells for regenerative therapies. The advantage of the use of iMSCs is that they can be generated from well-characterized and banked iPSCs with known HLA types. Another advantage of iMSCs over their native counterparts is that iMSCs have been characterized as rejuvenated MSCs.^14^ Although they are derived from pluripotent cells (which are by definition tumorigenic), iMSCs themselves are free from the risk of tumor formation since they do not express oncogenic pluripotency-associated genes such as OCT4.^15^ Moreover, iMSCs outperformed native MSCs in the treatment of multiple sclerosis in a rodent model.^16^ More importantly, iMSCs have been successfully used in vivo to improve bone regeneration by their direct differentiation into bone cells and their recruitment of host cells in a radial defect model in mice.^10^ The aim of this study was to evaluate the feasibility and impact of the use of a composite made of human iMSCs and calcium phosphate granules (CPG) for bone regeneration compared with that of a previously investigated autograft treatment, a composite made of autologous BMCs and CPG, and CPG alone in a critical-size long bone defect in mini-pigs under weight-bearing conditions in the early phase of bone healing. To the best of our knowledge, this investigation is the first to evaluate the regenerative potential of human iMSCs in a large animal model under the aforementioned conditions.

## Results

### Reprogramming of HFFs into iPSCs

**Fig. 1:**
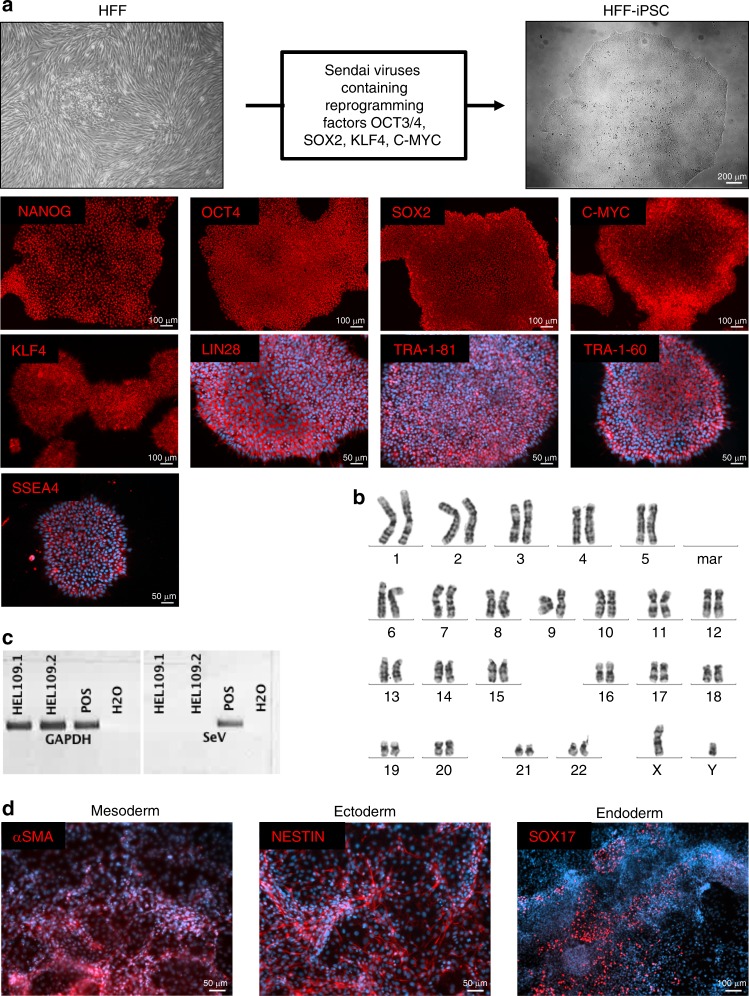
Characterization of HFF-derived iPSCs. **a** Protocol used for the generation of HFF-iPSCs and the confirmation of pluripotency marker expression by immunofluorescence-based detection. **b** Karyotype of the HFF-iPSCs. **c** Viral vector dilution PCR. **d** Evaluation of embryoid body formation by using immunofluorescence-based staining. Cell nuclei are stained using Hoechst stain (blue)

Human fetal foreskin fibroblasts (HFFs) were used to generate iPSCs by employing Sendai viruses encoding the reprogramming factors OCT3/4, SOX2, KLF4, and C-MYC. The HFF-iPSCs grew as colonies and expressed the pluripotency-regulating transcription factors OCT4, SOX2, NANOG, C-MYC, and KLF4 in addition to LIN28, SSEA4, TRA-1-60, and TRA-1-81 (Fig. 1a). A normal human male karyotype (46, XY) with no chromosomal aberrations was observed (Fig. 1b), and the absence of viral DNA was confirmed by PCR (Fig. 1c). Embryoid body formation assays demonstrated the capability of the HFF-iPSCs to differentiate into mesoderm (αSMA), ectoderm (NESTIN) and endoderm (SOX17) (Fig. 1d).

### Characterization of the HFF-iMSCs

**Fig. 2:**
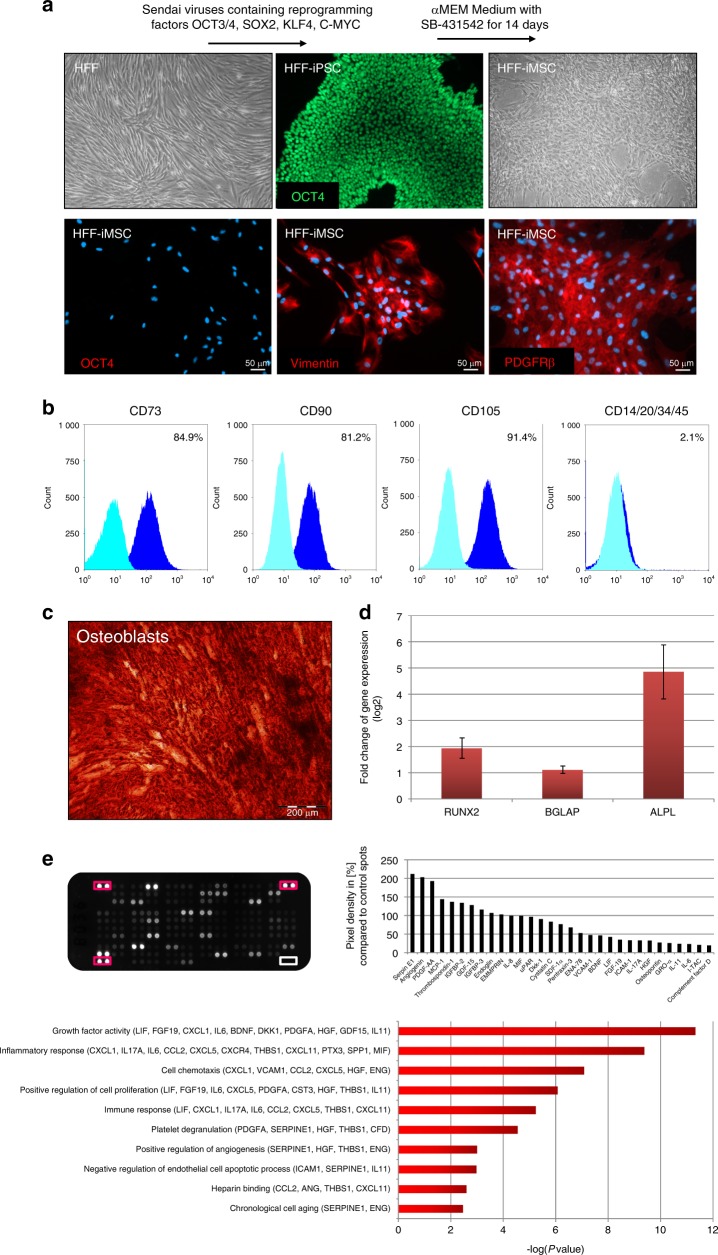
Properties of HFF-iPSC-derived iMSCs. **a** HFF-iMCS were analyzed with respect to their morphology and protein expression. The cell nuclei were stained with Hoechst. **b** Flow cytometric analysis using MSC cell surface markers (dark blue: specific cell surface markers; light blue: antibody isotype controls). **c** Alizarin Red S staining after osteogenic differentiation for 3 weeks. **d** Quantitative real-time PCR results for bone-related genes (in triplicate, normalized to the levels in untreated cells). **e** Cytokine membrane incubated with HFF-iMSC-conditioned media (left) and the background-corrected top 31 detected cytokines representing each of the selected associated GO terms; *P*-value < 0.05 (right)

The HFF-iPSCs were differentiated into HFF-iMSCs using a 14-day protocol that utilized the inhibition of the TGFβ pathway by SB431542.^17^ HFF-iMSCs showed a typical fibroblast spindle-shaped morphology and expressed the MSC markers PDGFRβ and Vimentin. Importantly, in contrast to the HFF-iPSCs, they were devoid of OCT4 expression (Fig. 2a). Cell surface marker analysis revealed that they exhibited a typical MSC immunophenotype by expressing CD73, CD90, and CD105 versus the hematopoietic markers CD14, CD20, CD34, and CD45 (Fig. 2b). The HFF-iMSCs were able to differentiate into adipocytes and chondrocytes (Fig. S1). More importantly, osteoblast differentiation in vitro was confirmed by Alizarin Red S staining of the emerged calcium deposits (Fig. 2c) and also by the upregulated expression of the bone-related genes *RUNX2, BGLAP*, and *ALPL* (Fig. 2d). The secretome of the HFF-iMSCs was investigated using a cytokine membrane assay able to detect 103 distinct cytokines. The top 31 secreted cytokines included serpin E1, angiogenin, PDGF-AA, and osteopontin, which are known to play an important role in skeletal regeneration processes. The associated GO terms “growth factor activity”, “cell chemotaxis” and “positive regulation of angiogenesis” imply the beneficial properties of these factors that are secreted by HFF-iMSCs (Fig. 2e).

### Transcriptome and STR analysis of HFF-iMSCs

**Fig. 3:**
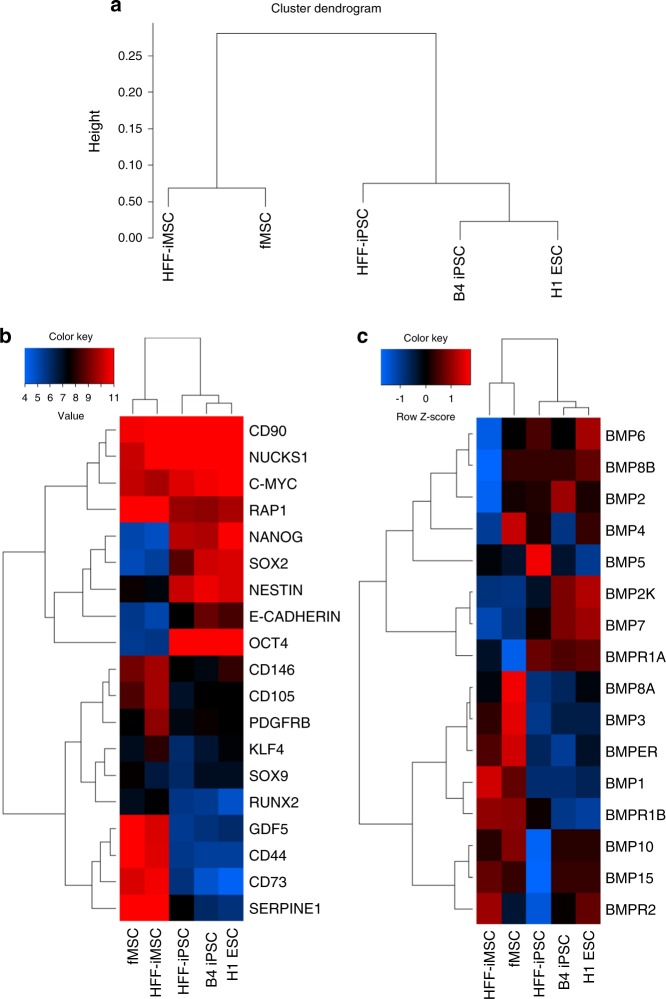
Microarray analysis of the HFF-iMSCs. **a** Cluster dendrogram of the HFF-iMSCs, fMSCs and pluripotent stem cells. **b** Heatmap depicting differential gene expression in HFF-iMSCs, fMSCs and pluripotent stem cells (iPSCs and ESCs). **c** Heatmap displaying the differential expression of BMPs and their corresponding receptors

The transcriptomes of the HFF-iMSCs were compared with the transcriptomes of iPSCs, ESCs, and fMSCs by microarray analysis. Cluster analysis revealed two groups: one that included the pluripotent cells, including the HFF-iPSCs, B4-iPSCs, and H1-ESCs, and the other that included the MSCs, HFF-iMSCs and primary fetal MSCs (fMSCs) (Fig. 3a). The expression of the MSC marker genes *CD44, CD73, CD105, CD146*, and *PDGFRβ* was confirmed. Notably, the expression of the key pluripotency-associated transcription factors, *OCT4, NANOG*, and *SOX2* was downregulated in HFF-iMSCs compared to iPSCs and ESCs (Fig. 3b). Furthermore, transcriptome analysis revealed the expression of several BMPs and their corresponding receptors (Fig. 3c). Pearson correlation analysis of the transcriptome data showed a high correlation of HFF-iMSCs with fMSCs (*R*^2^ value 0.947) and a low correlation with pluripotent stem cells (Fig. S2). Short-tandem-repeat (STR) analysis of the parental HFFs, HFF-iPSCs, and HFF-iMSCs verified their common genetic background (Fig. S3).

### Multilevel analysis of bone defect regeneration

CPG loaded with HFF-iMSCs were transplanted into a critical-size bone defect in the proximal tibia (see Fig. S5) in 8 mini-pigs. The results of the multilevel analysis were compared to those of the analysis of the three previously described standardized experimental groups, CPG, BMC + CPG and Autograft,^4^ which were used as controls within the present study to reduce the unnecessary sacrifice of mini-pigs.

### Histological evaluation

**Fig. 4:**
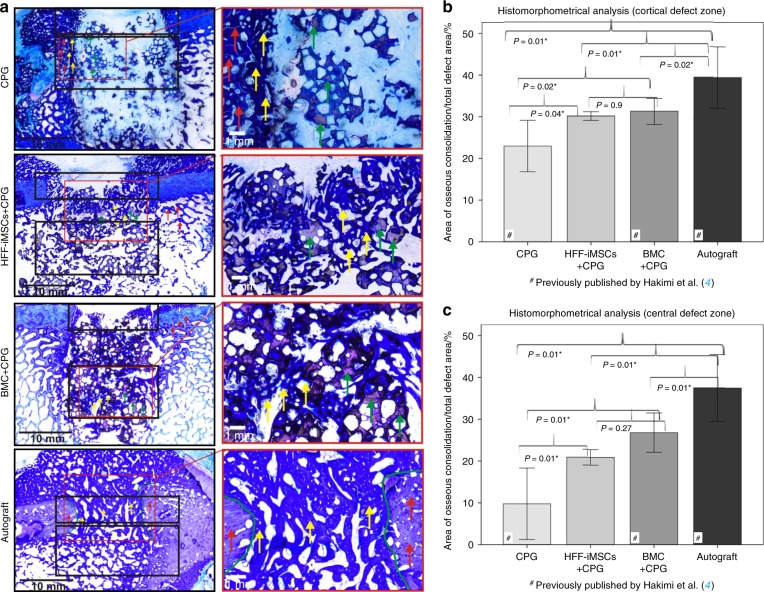
Histomorphometrical and radiological analysis of regenerated bone defects after 6 weeks. **a** Representative histological bone sections from all experimental groups after a regeneration period of six weeks (left: overview image depicting the cortical (upper black box) and central defect zones (lower black box); right: detailed image); yellow arrows: newly formed bone (royal blue); red arrows: former cortical bone (purple); green arrows: nonresorbed remnants of the CPG. **b** Histomorphometrical evaluation of the cortical defect zone. **c** Histomorphometrical evaluation of the central defect zone. The results for the CPG, BMC + CPG and Autograft groups were previously published by our group.^4^ *n* = 8 for each group; values are presented with the standard deviation)

Defect closure in all 4 experimental groups was confirmed by radiographic analysis after 6 weeks of regeneration (Fig. 4a). According to the histomorphometrical analysis of the cortical area, new bone formation was significantly lower in the CPG group compared to that in the CPG + HFF-iMSC (*P* < 0.04), CPG + BMC (*P* < 0.02), and Autograft groups (*P* < 0.01). The area of new bone formation was 23% ± 6.2% in the CPG group and 31.2% ± 3.1% in the BMC + CPG group, and in the HFF-iMSCs + CPG group, the defect filling area was 30.1% ± 1%. This, however, was significantly inferior (vs. HFF-iMSCs + CPG *P* < 0.01, vs. BMC + CPG *P* < 0.02) compared to the mean osseous consolidation of 39.4% ± 7.4% observed in the Autograft group. No significant differences were observed between the HFF-iMSCs + CPG and BMC + CPG groups (*P* = 0.9).

Similar results were observed in the central defect area. A mean osseous consolidation of 9.8% ± 8.5% was observed when using CPG alone. This was significantly lower than that in all other groups (*P* < 0.01). The area of new bone formation in the central defect area of the HFF-iMSC + CPG group was 20.9% ± 1.9%, and it was 26.8% ± 4.7% in the BMC + CPG group and 37.4% ± 8% in the Autograft group. The values observed in the Autograft group were significantly greater than those observed in all other groups (*P* < 0.01). No significant differences were observed between the HFF-iMSCs + CPG and the BMC + CPG group (*P* = 0.27). Relevant histological signs of inflammation caused by the grafting materials/cells were not found in any of the specimens (Fig. 4a–c).

### Multidetector computed tomography (MDCT) volumetry

**Fig. 5:**
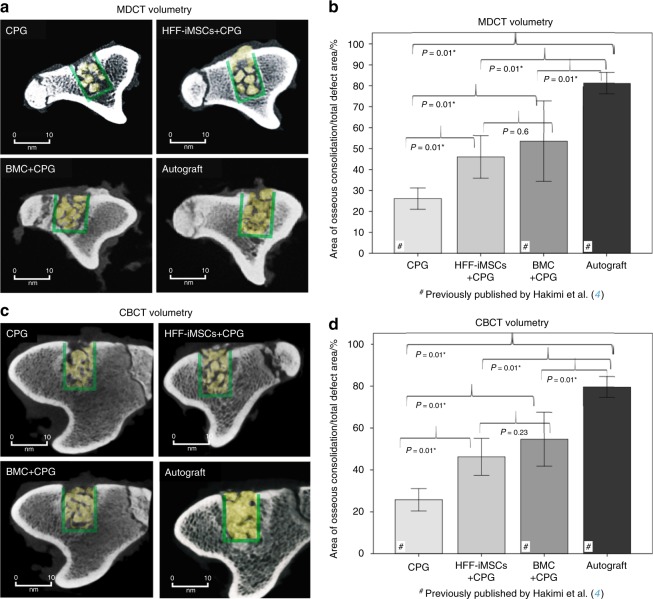
Radiological analysis of regenerated bone defects after 6 weeks. **a** Axial MDCT volumetry images of the tibial defect; only areas with a density >500 HU are indicated (yellow area). The green circled area represents the defect zone. **b** MDCT volumetry evaluation of bone defect consolidation. **c** Axial CBCT volumetry images of the tibial defect; only areas with a density >2 350 HU are indicated (yellow area). The green circled area represents the defect zone. **d** CBCT volumetry evaluation of bone defect consolidation. The results for the CPG, BMC + CPG and Autograft groups were previously published by our group.^4^ *n* = 8 for each group; values are presented with the standard deviation

The mean extent of bone defect consolidation was 46% ± 10.1% in the CPG + HFF-iMSCs group, 53.5% ± 19.1% in the BMC + CPG group, and 81.1% ± 5.1% in the Autograft group. The volume of new bone formation within the defect was 26.1% ± 5.1% in the CPG group, which was significantly inferior compared to that in all other groups (*P* < 0.01). Concerning the volume of new bone formation, the HFF-iMSCs + CPG group was similar to the BMC + CPG group (*P* = 0.6), and the volume in both groups was significantly lower compared to that of the Autograft group (*P* < 0.01) (Fig. 5a, b).

### Cone-beam computed tomography (CBCT) volumetry

CBCT volumetry analysis of the mean osseous consolidation in the HFF-iMSCs + CPG group, the BMC + CPG group, and the Autograft group found a volume of new bone formation of 46.3% ± 8.8%, 54.7% ± 12.8%, and 79.5% ± 5%, respectively, in the defect area. The volume of new bone formation was significantly greater in the Autograft group compared to the HFF-iMSCs + CPG and BMC + CPG groups (*P* < 0.01). There were no significant differences between the HFF-iMSCs + CPG and BMC + CPG groups (*P* = 0.23). The reconstructed area in the CPG group was 25.8% ± 5.3% and was significantly lower compared to that in all other groups (*P* < 0.01) (Fig. 5c, d).

## Discussion

Within the limitations of this translational study, it could be demonstrated that a composite containing human HFF-iMSCs and CPG was potent in inducing bone regeneration in the early phase of bone healing during the first six weeks. This in vivo model approximates the preclinical setting, as the species-specific (mini-pig) bone regeneration capacity (1.2–1.5 mm per day) mimics that found in humans under normal anatomical and physiological conditions.^18^ In current clinical practice, the treatment of large bone defects and bone nonunion in humans relies on bone grafting.^19^ Bone marrow-derived MSC (BM-MSC) transplantation has been proposed as a possible alternative.^20–22^ However, the scarcity of bone grafts, donor-associated disorders, the invasiveness of BM-MSC collection and immune rejection are possible drawbacks. Recently, the craniofacial bone regeneration potential of autologous MSCs was reported in small-animal models.^9^ For long bone reconstruction in sheep and for human facial remodeling, the utility of BM-MSCs has also been demonstrated in combination with scaffolds and BMP7.^21,23^ However, to date, only a limited number of studies have implemented preclinical animal models for weight-bearing long bone defect regeneration.

In this study, human iMSCs were used, as it has been reported that the differentiation and proliferation potential of primary MSCs in vitro diminish upon aging.^12^ In contrast, iMSCs generated from iPSCs or ESCs, when compared to BM-MSCs, show a similar phenotype but have a longer life span.^24^ Human iMSCs are characterized by a superior molecular signature in terms of rejuvenation compared to adult MSCs.^14^ Furthermore, iMSCs are currently in use in a human phase 1 clinical trial of GvHD [NCT02923375].

In this investigation, HFFs were used for iPSC generation by employing nonintegrating Sendai viruses; thus, the resulting HFF-iPSC line was devoid of transgenes. The HFF-iPSCs were positive for the Yamanaka factors^25^ and were chromosomally normal. The HFF-iMSCs that differentiated from the HFF-iPSCs expressed typical MSC markers, such as CD105 and Vimentin, and were devoid of the pluripotency-associated markers OCT4 and NANOG; subsequently, they did not result in tumor formation, as also observed in our earlier study.^15^ However, to ensure patient safety, long-term studies need to be conducted to evaluate the probability of tumor formation.

Upon osteogenic differentiation in vitro, the HFF-iMSCs showed a high rate of calcification and expressed high levels of the key transcription factor *RUNX2*^26^ and other important bone-related genes, including *BGLAP* and *ALPL*. Furthermore, they secreted immune-modulatory and osteo-regenerative cytokines such as PDGF-AA and osteopontin, thus avoiding the necessity for additional supplementation in cell culture. It was previously reported that BM-MSC supernatants induce the expression of bone-related genes, such as *BGLAP* and *ALPL*,^27^ and iPSC-MSCs have been shown to inhibit caspase activity in T-cells by producing TGF-β.^28^

To attain significance and clinical impact, we used 32 skeletally mature mini-pigs that were split into four groups of eight. Of these four groups, three groups were previously described by our group^4^ and were used as references in the present study: the autologous spongiosa group was used as the gold standard autograft control, the autologous BMC (bone marrow concentrate) combined with CPG group served as the positive control and the CPG alone group was used as the negative control. For the present study, HFF-iMSCs loaded on calcium phosphate granules were transplanted into a surgically induced bone defect in 8 mini-pigs. In all cases, even though no immunosuppression was administered to the pigs, obvious postoperative events, such as inflammatory reactions were not observed histologically. By applying histomorphometric, MDCT and CBCT analyses, we observed the successful reconstruction of bone mass. To mimic the surgical procedures used in humans, the implantation and explantation were performed by an expert group of orthopedic surgeons according to standard clinical protocols used for human patients.

In the current study, a minimal number of cells (1 × 10^6^) was transplanted to simulate the conditions typical to clinical settings, where the feasibility of long-term in vitro cell expansion is limited due to the amount of restricted time available for the treatment of the patient. Radiologically and histomorphometrically, the transplantation of the HFF-iMSCs loaded on CPG led to significantly better osseous consolidation in the central and cortical defect zones compared to that obtained with the use of CPG alone. Furthermore, in comparison with the composite of autologous BMC + CPG, no significant differences could be found in the cortical and central defect areas. These results are noteworthy since BMCs contain platelets and growth factors in addition to bone marrow MSCs,^4,29^ whereas the iMSCs were transplanted without the addition of exogenous factors. Furthermore, autologous BMC was used when the iMSCs were of human origin, and no administration of immune suppression was necessary. As expected, both radiologically and histomorphometrically, autologous bone transplantation resulted in the highest rate of new bone formation, which was significantly higher compared to that observed in all other groups. In a rat model of critical-size cranial defects, human iMSCs performed comparably to human MSCs (bone marrow and umbilical cord) and showed 2.8-fold improved regeneration compared to calcium phosphate cement alone after 12 weeks.^30^ Another study demonstrated that human iMSCs contributed to substantial bone formation and produced a significantly better outcome than primary human BM-MSCs in a mouse radial defect model.^10^ In addition to the use of iMSC in in vivo studies, a protocol has been described for generating bone substitutes by the incubation of iMSC-loaded scaffolds in a perfusion bioreactor system with the aim of using these in personalized bone tissue engineering in the near future.^31^ In addition to the use of different scaffold materials, the supplementation of BM-MSCs with growth factors such as BMP-7 has been used to stimulate osteogenic reconstruction.^23^

Moreover, the transplantation of primed or osteogenic-differentiated MSCs into bone defect models has also been reported.^9,32^ In the current study, human iMSCs were transplanted without the addition of growth factors and at their full multipotent capacity to enable HFF-iMSCs to function as immunosuppressors and inducers of regeneration (paracrine effects) and to directly contribute to bone formation. The successful outcome of the transplantation of the composite of HFF-iMSCs and micro- and macroporous calcium phosphate granules (CPG) may have been due to the characteristics of the specific scaffold material used. An in vitro compatibility test of HFF-iMSCs and CPG (see Fig. S4) showed that HFF-iMSCs can be absorbed by CPG and remain alive and functional within the scaffold material. The CPGs that were utilized are composed of carbonated, calcium-deficient apatite calcium phosphate. They mimic human bone material more closely than HA or TCP cement.^33^ Another advantage of CPG is the presence of both small and large pores, which enable the three-dimensional ingrowth of newly formed bone mass into the scaffold material.^34^ Furthermore, the use of granules leads to a faster resorption rate in vivo when compared to the use of compact blocks of identical material.^35^ The reported high wicking capability of CPG^33^ enabled the transplantation of iMSCs into bone defects after absorption into the scaffold material. CPG was used as a scaffold material because it has been shown to have beneficial effects on bone reconstruction.^36^ Furthermore, CPG have been used successfully as a scaffold material in combination with other fluid osteoinductive substances, such as platelet-rich plasma (PRP), BMC and a combination of both in the animal model used by our group.

**Fig. 6:**
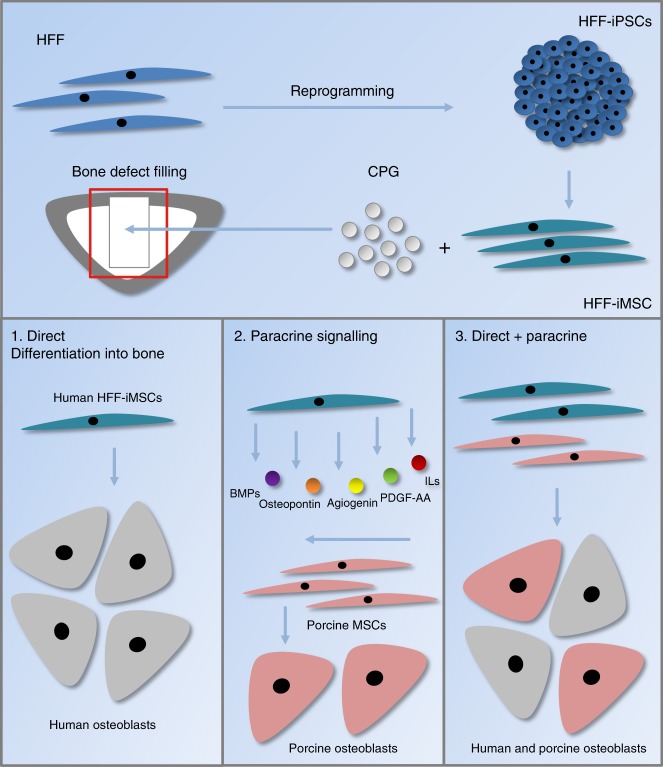
Possible modes of action of the HFF-iMSCs. We propose three potential mechanisms whereby HFF-iMSCs contribute to the regeneration of critical-size bone defects. 1: Niche-induced differentiation into human osteoblasts; 2: paracrine signaling-induced regeneration by the activation and recruitment of resident stem cells; 3: a combination of niche-induced differentiation and paracrine signaling

One limitation of our study is the use of CT to evaluate the newly formed bone; this method cannot discriminate between the bone substitute material CPG and newly formed bone mass because both structures have comparable densities. However, the radiological and histomorphometrical analyses used in this study represent well-established evaluation methods that have been used by our group in previous studies with this type of animal model.^4,37–39^ Under these circumstances, a high correlation between the results of the histomorphometrical analysis and the two independent CT analyses confirmed the reliability of our system for evaluating osseous consolidation noninvasively, as previously shown.^4^ Furthermore, this investigation did not make an exact determination of the molecular mechanism(s) associated with bone regeneration and did not evaluate whether neo-bone formation was a direct effect of the implanted cells or the effects of secreted factors, such as immune modulation and pro-angiogenic signaling factors. Since the factors secreted from MSCs and iMSCs have been described as significantly influencing the therapeutic effect via interaction with immune cells,^40,41^ immune modulation and paracrine signaling might have played a pivotal role, as indicated by the analysis of the secretome of the HFF-iMSCs that revealed the presence of serpin E1, angiogenin, PDGF-AA and osteopontin; in addition, the transcriptome analysis showed the expression of several BMPs associated with bone regeneration.^23,42^ Furthermore, it must be taken into account that the BMP2/4 and NF-κb signaling pathways play important roles in the paracrine pathways involved in the bone regeneration process by regulating the secretory profile of MSCs.^43,44^ Key components of these pathways, RAP1 and NUCKS1, were shown to be expressed by HFF-iMSCs (see Fig. 3b). We postulate that there are potential mechanisms whereby HFF-iMSCs might contribute to the regeneration of critical-size bone defects, including (i) their niche-induced differentiation into human osteoblasts, (ii) their paracrine signaling-induced regeneration via the activation and recruitment of resident porcine stem cells, and (iii) a combination of differentiation and paracrine signaling (Fig. 6). Ultimately, iMSC tracing experiments will be required to investigate the homing/chemotactic effects of iMSCs and the efficiency of their expansion in vivo in subsequent studies.

The positive effects of the human HFF-iMSC composite in the early phase of bone healing could possibly lead to follow-up experiments that would be conducted for longer than 6 weeks. Additionally, the monitoring of defect healing, biomechanical evaluation and an increase in the numbers of transplanted cells could be of use in future studies.

Using the iPSC approach, it is possible to generate HLA-matched iMSCs for treating distinct bone defects, thus reducing the need for patient-derived BMCs as well as BM-MSCs. Human HFF-iMSC engrafting was shown in vivo to lead to the formation of new bone six weeks posttransplantation, thus demonstrating the usefulness of iMSCs for the future treatment of large bone defects. However, clinical applications will require significant improvements to optimize applicability, ensure patient safety and increase the in-depth understanding of the basic biomolecular processes involved in regeneration and the long-term posttransplantation effects.

## Materials and methods

### Generation of HFF-iPSCs

Human fetal foreskin fibroblasts were reprogrammed at the Biomedicum Stem Cell Center (Helsinki, Finland) using Sendai virus vectors encoding the reprogramming factors OCT3/4, SOX2, KLF4, and C-MYC. The reprogramming and culture of the iPSCs were carried out under feeder-free conditions using Matrigel (Becton Dickinson, Heidelberg, Germany) and E8 medium (Thermo Fisher Scientific, Darmstadt, Germany) or StemMACS IPS BREW medium (Miltenyi Biotec, Bergisch Gladbach, Germany). The clearance of the Sendai virus was confirmed by PCR; we referred to the HFF-derived iPSCs as HFF-iPSCs.

### Embryoid body formation

The pluripotency of the iPSCs was confirmed by an embryoid body assay demonstrating the ability of the iPSCs to spontaneously differentiate into cell types representative of the three germ layers (ectoderm, mesoderm, and endoderm) as described previously.^45^ Please refer to Table S1 for a list of the antibodies used. Further details are provided in the Supplementary Methods.

### Karyotyping of the HFF-iPSCs

The karyotype analysis was carried out by the Institute of Human Genetics and Anthropology, Heinrich-Heine-University, Düsseldorf, Germany.

### Generation of HFF-iMSCs

iMSCs were generated from HFF-iPSCs by using a modified version of an already published protocol^17^ that utilized the TGFβ pathway inhibitor SB 431542 to facilitate epithelial to mesenchymal transition. The iPSCs were cultured under feeder-free conditions on Matrigel (Becton Dickinson, New Jersey, USA) using human StemMACS iPS BREW XF medium (Miltenyi Biotec). When the cell layer covered ~50% of the well, the medium was switched to α-MEM (alpha-modified minimum essential medium; Sigma-Aldrich, Taufkirchen, Germany) supplemented with 10% FBS, 1% GlutaMAX and 1% P‧S^–1^ without basic fibroblast growth factor. This medium was supplemented with 10 µmol·L^–1^ SB 431542 (Miltenyi Biotec). For 14 days, the cell culture medium was changed daily. The cells were harvested using TrypLE Express and were reseeded onto uncoated culture dishes in α-MEM without SB 431542 supplementation. After several passaging steps, the cells were characterized as iMSCs. The general cell culture reagents were obtained from Gibco (Thermo Fisher Scientific, Darmstadt, Germany).

### Transcriptome analysis

The microarray analysis was performed by using the PrimeView Human Gene Expression Array platform (Affymetrix, Thermo Fisher Scientific). The data are accessible online via the National Center of Biotechnology Information (NCBI) Gene Expression Omnibus. Further processing of the nonnormalized bead summary data was performed using R/Bioconductor software^46^ with the affy package (http://bioconductor.org/packages/release/bioc/html/ affy.html).^47^ After background correction, the values were converted to a logarithmic scale (to base 2), and normalization was performed using the robust multi-array average method. Ethically approved fetal MSCs (kindly provided by Prof. Richard O.C. Oreffo, University of Southampton-UK) were used as the reference cells.

### Flow cytometry

The MSC Phenotyping Kit Human (# 130-095-198) from Miltenyi Biotec was used to identify the cell surface profile of the HFF-iMSCs according to the manufacturer’s instructions. The labeled cells were analyzed using a FACSCanto from BD Biosciences (Heidelberg, Germany). The histograms were generated using Summit 4.3.02 software. The protocol used for cell preparation can be found in the Supplementary Methods.

### Demonstration of the multipotency of HFF-iMSCs

The differentiation of HFF-iMSCs into adipocytes, chondrocytes, and osteoblasts was performed with the STEMPRO Adipogenesis, Chondrogenesis and Osteogenesis Differentiation Kit (Thermo Fisher Scientific). The differentiation was carried out for 3 weeks with media changes every 2–3 days. After this period, the cells were fixed with PFA and stained as described previously.^48^ The staining procedures are described in the Supplementary Methods.

### Secretome analysis of the HFF-iMSC-conditioned media

The molecules secreted from the HFF-iMSCs were identified using the Proteome Profiler Human Cytokine Array Panel A (R&D Systems), which consists of a membrane with 103 different spotted antibodies, according to the user manual. For the detection of cytokines, 1.5 mL of conditioned medium from HFF-iMSCs was incubated on the cytokine membrane. The membrane was analyzed by detecting the emitted chemiluminescence. The pixel density of each spot, representing the amount of bound cytokine, was analyzed using ImageJ software. The value of the negative control was subtracted from all other values. Then, every value was divided by the mean of the values of the reference spots and multiplied by 100 to determine the percentage value in comparison to the reference spots.

### Immunofluorescence staining

The cells were stained as described previously.^48^ Please refer to the Supplementary Methods for a detailed description. The list of primary antibodies used can be found in the Supplementary Material (Table S1).

### Real-time reverse transcriptase-polymerase chain reaction (qRT-PCR)

Real-time quantitative PCR was performed for each technical triplicate using the Power SYBR Green Master Mix (Life Technologies) with a VIIA7 instrument (Life Technologies). The program used consisted of the denaturation of the samples at 95 °C for 2 min, followed by 40 cycles of amplification (30 s of denaturation at 95 °C, annealing at the primer-specific temperature (57 °C–63 °C) for 30 s, and extension at 72 °C for 30 s). The primers were purchased from MWG, and the specific sequences, as well as the amplicon sizes, are provided in the Supplementary Material (Table S2). For the analysis of the qRT-PCR data, the housekeeping gene encoding ribosomal protein L37A was used to normalize the values of the tested genes. The expression levels were calculated using the ΔΔCT method and are shown as the mean value with the standard error of mean. The procedures used for RNA isolation and cDNA synthesis are described in the Supplementary Methods.

### Bone defect model and cell transplantation

All animals were handled in compliance with the guidelines for the care and use of animals at our institution and in accordance with the EU Directive 2010/63/EU for animal experiments. Approval from the regional ethics committee for animal experiments (LANUV NRW, Recklinghausen, Germany) was obtained (Permit Number: 84-02.04.2015.A042). In this study, 8 female Goettingen mini-pigs (aged 20–28 months, weight 24 kg–35 kg) were used. Based on previous studies performed by our group utilizing a similar animal model and an a priori power analysis, a sample size of 8 was determined to have a power of 80%, and a *P*-value of 0.05 denoted significance.^49^

The animals were randomly assigned to one of the study groups (each group consisted of eight Goettingen mini-pigs). All defects were filled entirely using a volume of 2.4 cm^3^. In the CPG group, the defects were filled with calcium granules alone, and in the BMC + CPG group, the defects were filled with autologous BMCs in combination with CPG. In the autograft group, the defects were filled with autologous bone harvested from the iliac crest. For this, the iliac crest was exposed, and a Kirschner guide wire (K-wire) was inserted. Using a cannulated reamer placed on the guide wire, cancellous bone was harvested. The results from these 3 groups have been reported by our group^4^ and were used as controls in the present study to avoid the loss of additional animals and for ethical reasons. Preliminary experiments were carried out by our group in which the same defect was created in the proximal tibia of four mini-pigs without the addition of any filling material. Because of a proximal tibia fracture that occurred within 3 days after operation, all of these animals had to be sacrificed prematurely.^50^ Therefore, the defect model used in the current study fulfills the criteria of a critical-size defect model. To prevent the unnecessary sacrifice of additional animals and for ethical reasons, the present study was carried out without a no treatment control.

In accordance with the animal model developed by our group,^49^ a cylindrical defect of 11 mm diameter and 25 mm depth was created in the right proximal tibia medially using a cannulated reamer (Aesculap AG & Co. KG, Tuttlingen, Germany). In the CPG, BMC + CPG and HFF-iMSCs + CPG groups spherical, micro- and macroporous (micro: 2 µm–10 µm; macro: 150 µm–550 µm), carbonated, and apatite calcium phosphate granules 2 mm–4 mm in size (Calcibon® Granules, Biomet Deutschland GmbH, Berlin, Germany) were used.

All surgical procedures were performed with single anesthesia by the same experienced surgeon under strict aseptic conditions. Further methodological details are described in the Supplementary Methods. Using a medial approach in the right proximal tibia, the defect was created 10 mm distal to the joint line and 12 mm anterior to the most posterior aspect of the tibia. In the BMC group, bone marrow was harvested from the iliac crest, and mononuclear cells were concentrated to generate bone marrow concentrate (BMC) using a point-of-care device (MarrowStim® mini concentration system, Biomet Biologics, Inc., Warsaw, Indiana, USA) as described previously.^4^ In the HFF-iMSCs + CPG group, the CPG were soaked with a mixture of 1 × 10^6^ HFF-iMSCs (passage numbers 5, 7, and 9) for five minutes prior to implantation. The soft tissues were closed in layers.

Postoperatively, all animals were allowed to bear their full weight. At 42 days after the procedure, the animals were sacrificed using 3% sodium pentobarbital (Eutha 77, Essex Pharma GmbH, München, Germany). The proximal tibia was harvested by a sharp dissection tool and fixed in 10% neutral buffered formalin solution for 14 days. Figure S5 shows a schematic of the bone defect.

### Statistical analysis

The statistical analysis was performed using SPSS software (version 21.0, SPSS Inc., Chicago, IL, USA). The mean values and standard deviations were calculated. The outcome measures of the radiological and histomorphometrical evaluations were examined by one-way analysis of variance (ANOVA). Differences between the independent variables were checked with post hoc tests [Tukey's HSD (honestly significant difference) test]. Significance was defined at a *P*-value < 0.05.

### Multidetector computed tomography (MDCT)

Using a 64-detector row CT scanner (SOMATOM Sensation Cardiac 64, Siemens Medical Solutions, Germany), radiographic analysis was performed as described previously.^39^ In brief, volumetric measurements were performed with respect to density in Hounsfield units (HU) according to axial images. A threshold value of 500 HU was defined for osseous consolidation, and the defect volume was measured three times at different HU ranges:

(i) Overall size of the defect: measured by including all pixels with an density between −100 and +3 000 HU.
(ii) Areas of consolidation: measurement of pixels with densities between 500 and 3 000 HU.
(iii) Nonconsolidated areas: measurement of all pixels with densities between −100 and 500 HU.

### Quantitative cone-beam CT (CBCT) volumetry

Using a CBCT scanner with a flat panel detector (PaX-Duo3D, Vatech, Korea), images were obtained as described previously.^38^ The bone defect volume and extent of new bone formation were evaluated quantitatively using DICOM viewer (Osirix Imaging Software, 64-Bit extended version, Pixmeo, Geneva, Switzerland). With respect to the density values, the volumetric measurements were performed after semiautomatic selection and by marking pixels with predefined density values on the axial images. Based on the mean density values of cortical and trabecular bone, a threshold value of 2 350 was defined for bone consolidation, and volumetric measurements of the defect were performed three times with three different settings:

(i) Overall size of defect: measured by including all pixels in the outlined defect
(ii) Areas of consolidation: measurement of pixels with densities >2 350
(iii) Nonmineralized areas: measurement of all pixels with densities <2 350.

The relative extent of bone regeneration and the absolute volumes of bone consolidation were determined.

### Histological preparation of the bone segments

For nondecalcified sectioning, all specimens were dehydrated using an ascending series of graded alcohol and xylene prior to infiltration and embedding in methylmethacrylate. Serial sections were cut in the axial direction using a diamond wire saw (Exakt®, Apparatebau, Norderstedt, Germany). Before staining, the toluidine blue-stained sections were ground to a final thickness of approximately 50 µm.

### Histomorphometrical analysis

Two experienced investigators who were blinded to the experimental groups performed all histomorphometric analyses and microscopic observations as described previously.^49^ In brief, the areas of new bone formation (µm^2^) and the percentage of total new bone formation were measured in the cortical and central defect areas (see Fig. S5). After visual identification, the tissue type was determined manually and assigned a color on three sections from each specimen. Based on this, the areas of newly formed bone, connective tissue, and CPG were calculated according to the total bone defect area.

## Supplementary information

Supplementary Information.

## Data Availability

The HFF, HFF-iPSC and HFF-iMSC transcriptome data are accessible online via the National Center of Biotechnology Information (NCBI) Gene Expression Omnibus.
