# Supplementary material for: Human iPSC-derived iMSCs improve bone regeneration in mini-pigs
Source: Bone Res. 2019 Oct 24;7:32. doi: 10.1038/s41413-019-0069-4 (PMC6813363; doi:10.1038/s41413-019-0069-4)
Supplement: Supplementary file 1 — Supplementary Information. [file 41413_2019_69_MOESM1_ESM.docx]

**Supplementary Material**

**
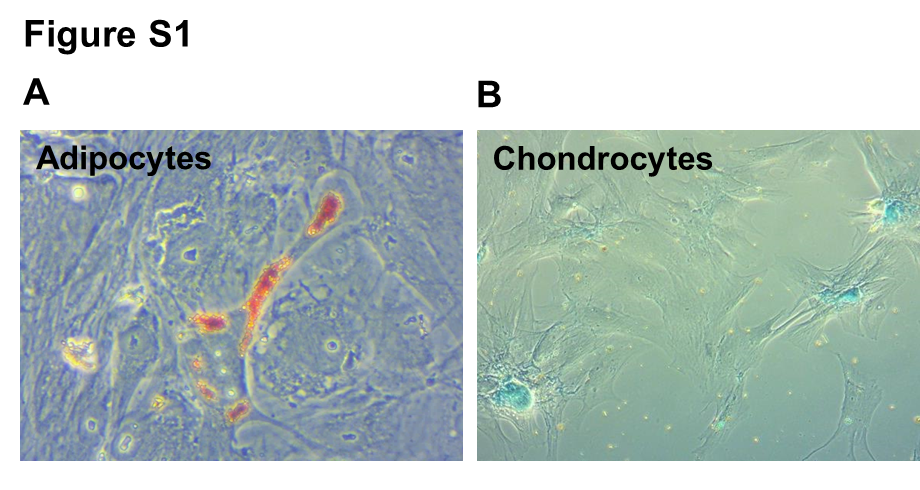
**

**Figure S1.** HFF-iMSC differentiation. (A) Oil Red O staining of evolved fat droplets generated by adipogenic differentiation. (B) Alcian Blue staining of proteoglycans within the chondrocyte clusters.

**
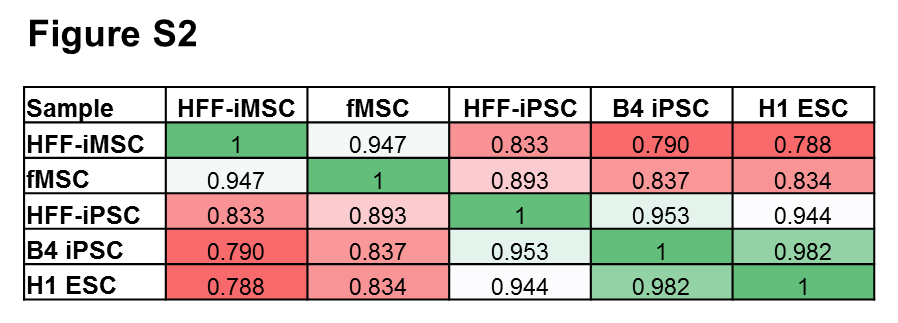
**

**Figure S2.** Pearson correlation values.


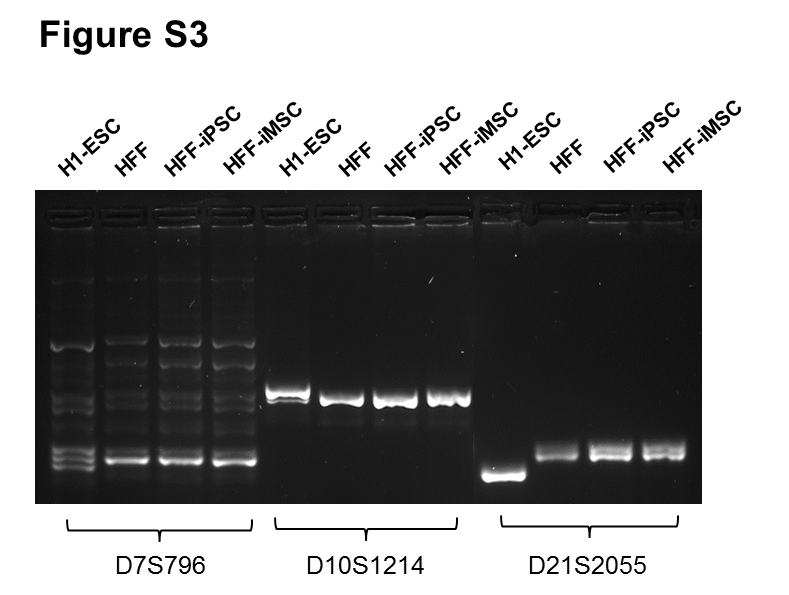


**Figure S3.** Short-tandem repeat analysis. Short-tandem repeat PCR of the HFFs, HFF-iPSCs and HFF-iMSCs with the primers D7S796, D10S1214 and D21S2055 confirmed their common genetic background and origin.

**
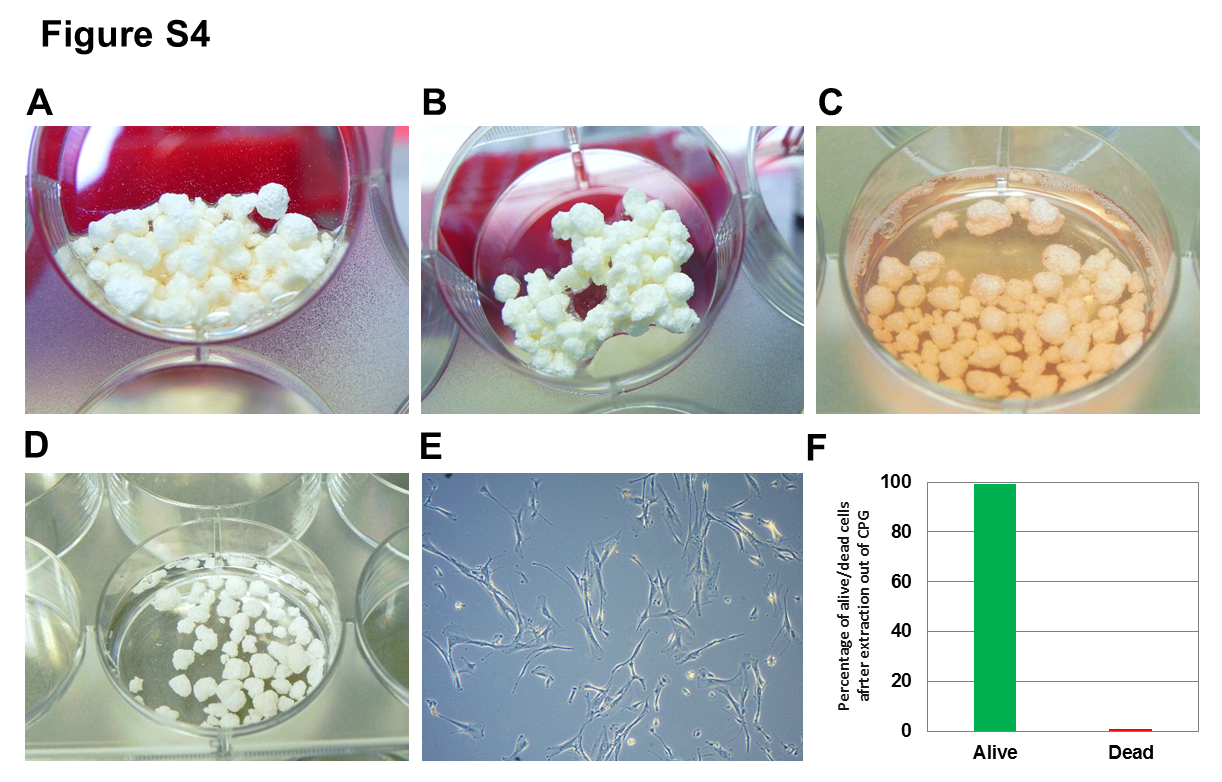
**

**Figure S4.** *In vitro* compatibility test of the HFF-iMSCs and CPG. (A) Loading of CPG with the HFF-iMSC suspension. (B) CPG after the absorption of the HFF-iMSC suspension. (C) Incubation of loaded CPG at 37°C in 5% CO_2_ for 24 h in MSC medium. (D) Extraction of HFF-iMSCs from CPG with TrypLE. (E) HFF-iMSC reattachment and growth on plastic dishes. (F) Plot of the percentage of live/dead cells after extraction from CPG.


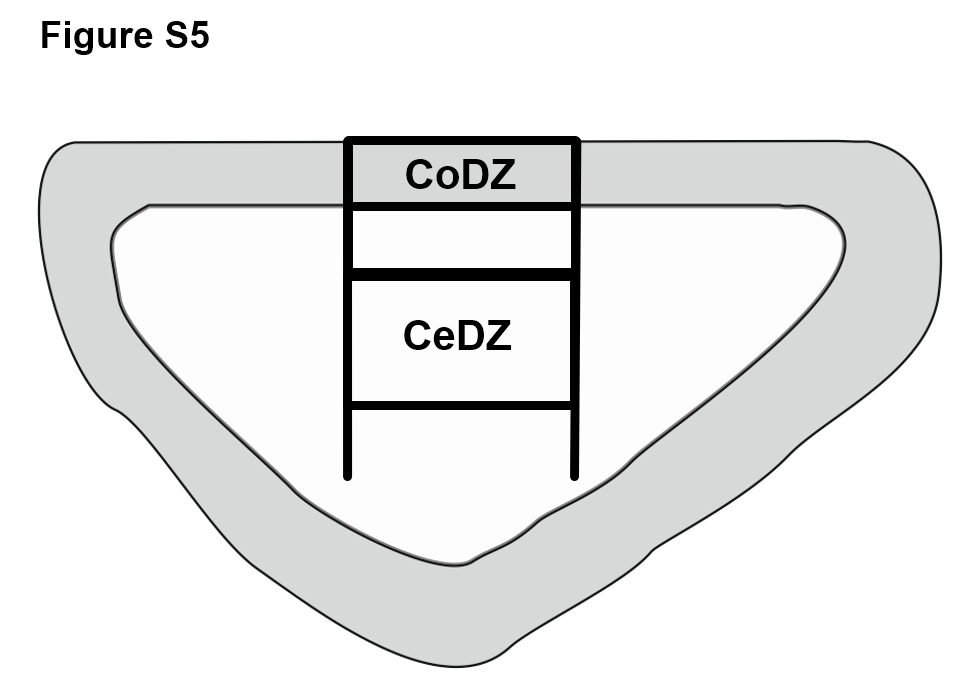


**Figure S5.** Schematic of a histological section from a critical-size bone defect in the proximal tibia. Histomorphometrical quantification of new bone formation was carried out in two distinct regions: the cortical defect zone (CoDZ) and the central defect zone (CeDZ). Adapted from Hakimi et al., 2014 (*4*).

**Table S1.** List of antibodies

| **Primary Antibody** | **Manufacturer** | **Dilution** |
| --- | --- | --- |
| OCT-4A (C30A3) rabbit mAb number 2840 | Cell Signaling Technology, USA | 1:400 |
| SSEA4 (MC813) mouse mAb number 4755 |  | 1:1000 |
| E-cadherin (24E10) rabbit mAb number 3195 |  | 1:200 |
| Vimentin (5G3F10) mouse mAb number 3390 |  | 1:200 |
| TRA-1-60 mouse mAb number 4746 |  | 1:1000 |
| TRA-1-81 mouse mAb number 4745 |  | 1:1000 |
| Rabbit anti-NANOG Cat# 4903S |  | 1:800 |
| Rabbit anti-SOX2 Cat# 3579S |  | 1:400 |
| c-Myc (D84C12) rabbit mAb #5605 |  | 1:400 |
| KLF4 Antibody #4038 |  | 1:400 |
| LIN28A (D84C11) XP® rabbit mAb #3695 |  | 1:400 |
| PDGF receptor β (28E1) rabbit mAb #3169 |  | 1:100 |
| CD133 PA2049 | Boster Bio, USA | 1:500 |
| C-Kit (H-300) rabbit polyclonal IgG | Tebu Bio, Germany | 1:200 |
| rb PODXL, sc33138 | Santa Cruz Biotechnology, USA | 1:200 |
| aSMA Cat# M0851, RRID:AB_2223500 | Dako (Agilent), USA | 1:1000 |
| Nestin Cat# N5413, RRID:AB_1841032 | Sigma-Aldrich, USA | 1:1000 |
| Mouse anti-Sox17 Cat# AF1924, RRID:AB_355060 | R and D Systems, USA | 1:250 |

**Table S2.** List of primers

| **Gene** | **Primer Sequences** |
| --- | --- |
| RUNX2 | F1: CAGACCAGCAGCACTCCATA R1: CAGCGTCAACACCATCATTC |
| BGLAP | F1: AAGGTGCAGCCTTTGTGTCC R1: GGCTCCCAGCCATTGATACA |
| ALPL | F1: CTATCCTGGCTCCGTGCTC R1: ACTGATGTTCCAATCCTGCG |
| D7S796 | F1: TTTTGGTATTGGCCATCCTA R1: GAAAGGAACAGAGAGACAGGG |
| D10S1214 | F1: ATTGCCCCAAAACTTTTTTG R1: TTGAAGACCAGTCTGGGAAG |
| D21S2055 | F1: AACAGAACCAATAGGCTATCTATC R1: TACAGTAAATCACTTGGTAGGAGA |

**Supplementary Methods**

**Staining of differentiated HFF-iMSCs**

For adipocyte staining, a 0.2% Oil Red O working solution was prepared by diluting the 0.5% stock solution with distilled water followed by filtration using Whatman paper. The medium was aspirated completely, and the cells were washed with PBS followed by 50% ethanol. The cells were stained with 0.2% Oil Red O solution for 10 min at room temperature. After two washes with 50% ethanol and distilled water, PBS was added to the cells to enable microscopic analysis. For chondrocyte staining, the cells were washed with PBS and then stained with 1% Alcian Blue solution (prepared in 0.1 N HCl) for 30 min at room temperature on a rocking platform. Afterwards, the cells were rinsed three times with 0.1 N HCl, covered with distilled water and analyzed by light microscopy. For osteoblast staining, the cells were rinsed with distilled water and stained with 2% Alizarin Red S solution (prepared in distilled water) for 30 min at room temperature on a rocking platform. Afterwards, the wells were washed three times with distilled water, and PBS was finally added to allow their visualization by light microscopy.

**Embryoid body formation**

Initially, the iPSCs were cultured on Matrigel using StemMACS IPS BREW medium. After reaching confluence, they were detached using PBS (without calcium and magnesium) and then cultured in T25 flasks in suspension culture in high-glucose DMEM containing 10% FBS, 1% P/S, 1% GlutaMAX and 1% NEAA. The emerged embryoid bodies were collected and placed into a gelatin-coated well. After 3-4 days, the embryoid bodies were fixed using 4% PFA and then stained with antibodies specific to each of the three germ layers (see Table S1).

**Immunofluorescence staining**

To block the nonspecific binding sites, blocking buffer was applied to fixed (4% PFA) cells for 2 h at RT. If intracellular proteins were to be stained, blocking buffer containing 10% normal goat serum (NGS, Sigma), 0.5% Triton X-100 (Carl Roth GmbH & Co. KG, Karlsruhe, Germany), 1% BSA (Sigma), and 0.05% Tween-20 (Sigma), all dissolved in PBS, was used. When extracellular proteins were stained, Triton X-100 and Tween-20 were omitted. Primary antibodies were incubated with the cells for 1 h at RT followed by 3 washing steps. Afterwards, the corresponding secondary antibodies (Thermo Fisher Scientific) and DAPI (Southern Biotech) or Hoechst 33258 dye (Sigma-Aldrich Chemie GmbH, Taufkirchen, Germany) were added to the cells, which were incubated for 1 h at RT in the dark. A fluorescence microscope (LSM700; Zeiss, Oberkochen, Germany) was used for imaging. Image processing was performed using ZenBlue 2012 software version 1.1.2.0. (Carl Zeiss Microscopy GmbH, Jena, Germany).

**Cell preparation for flow cytometry**

The cells were detached using TrypLE Express (Gibco) for 7 min at 37°C. After centrifugation at 300 g for 5 min, 200,000 cells were distributed into two 5 ml flow cytometry tubes in 2 ml phosphate buffered saline (PBS). The cells were then centrifuged for 10 min at 300 g, the supernatants were discarded and the pellets were resuspended in 100 µl PBS. The phenotyping cocktail (0.5 µl) was then added to the first of the two tubes. The cocktail contained CD14-PerCP, CD20-PerCP, CD34-PerCP, CD45-PerCP, CD73-APC, CD90-FITC, and CD105-PE antibodies. To the second tube, the isotype control cocktail was added. After 10 min of incubation at 4°C in the dark, the cells were washed with PBS and centrifuged at 300 g for 10 min. Afterwards, the pellets were resuspended in 100 µl paraformaldehyde (4% PFA) prior to flow cytometric analysis.

**RNA Isolation and cDNA Synthesis**

RNA isolation was performed using the Direct-zol RNA Miniprep Kit (Zymo Research, CA, USA) according to the manufacturer’s instructions. Complementary DNA (cDNA) synthesis with 500 ng of mRNA was performed using the TaqMan Reverse Transcription Kit (Applied Biosystems). The reaction mixture (20 μl per sample) included 7.70 μl H2O, 2 μl reverse transcriptase buffer, 4.4 μl MgCl2 (25 mM), 1 μl oligo(dT)/random hexamers (50 µM), 4 μl dNTP mix (10 mM), 0.4 μl RNase inhibitor (20 U/µl) and 0.5 μl reverse transcriptase (50 U/µl).

***In vitro* compatibility test for the HFF-iMSCs and CPG**

First, 2.4 cm^3^ CPG was loaded with 1100 µl of a suspension of 1x10^6^ HFF-iMSCs. After the cell suspension was completely absorbed by the CPG, the cell-loaded granules were covered with 5 ml αMEM medium and incubated at 37°C in 5% CO_2_ for 24 h. Afterward, the CPG were washed with PBS and incubated in TrypLE for 15 min at 37°C with repeated shaking. The live/dead ratio of the extracted cells was determined by Trypan blue staining, and the cells were reseeded into a plastic culture dish.

**Animal preparation for cell transplantation**

All mini-pigs were fasted for a minimum of 12 h before surgery, and peri-operative antibiotic prophylaxis was conducted by daily administration of 3.3 ml lincomycin (20% lincomycin, WDT, Garbsen, Germany) for three days prior to surgery. The animals were primed via intramuscularly administered sedation with 0.5 mg/kg atropine (atropine sulfate, B Braun, Melsungen, Germany), 5 mg/kg azaperone (Stresnil®, Janssen-Cilag GmbH, Neuss, Germany) and 10 mg/kg ketamine (Ketavet®, Pharmacia GmbH, Karlsruhe, Germany). Anesthesia was initiated with 0.5 g thiopental (Inresa Arzneimittel GmbH, Freiburg, Germany) and maintained via the inhalation of oxygen, nitrous oxide, and isoflurane. An infusion of 5% glucose solution (Delta-Select, Pfullingen, Germany), 10 ml inzolen (Koehler Chemie GmbH, Alsbach-Hähnlein, Germany), and 5 ml 2% lidocaine (lidocaine-HCl, B. Braun, Melsungen, Germany) was administered at a rate of during the procedure to maintain hydration and cardiac protection. By administering an intravenous injection of 0.4 mg/kg piritramide (Dipidolor®, Janssen-Cilag GmbH, Neuss, Germany) and 4.5 mg/kg carprofen (Rimadyl®, Pfitzer Pharma GmbH, Karlsruhe, Germany), intraoperative analgesia was maintained. For the treatment of postoperative pain, piritramide and carprofen were administered subcutaneously for three days.

**DNA fingerprinting analysis**

DNA isolation was performed using the QIAamp® DNA Mini Kit according to the manufacturer’s instructions. The analysis of short-tandem repeats (STR) was performed by PCR using specific primers (D7S796, D10S1214 and D21S2055). The primer sequences are provided in Supplementary Table 2. Each PCR contained 5 μl 1x Go-Taq G2 Hot Start Green PCR buffer, 4 μl 4 mM MgCl2, 0.5 μl dNTP mix (10 mM each), 1 μl forward primer (0.3 μM), 1 μl reverse primer (0.3 μM), 0.125 μl (0.625 U) Hotstart Taq polymerase (5 U/μl), and 100 ng genomic DNA. Water was added to adjust the final volume to 25 µl. A thermal cycler (PEQLAB, Erlangen Germany) was utilized for the PCR. The 5 min initial denaturation at 94°C was followed by 32 cycles consisting of a denaturation step at 94°C for 15 s, an annealing step at 60°C for 30 s, and an extension step at 68°C for 60 s. Gel electrophoresis (2.5% agarose gel) was used to detect the PCR amplification products. The primers that were used are listed in Table S2.
